# Effect of Dietary Composite Probiotic Supplementation on the Microbiota of Different Oral Sites in Cats

**DOI:** 10.3390/vetsci11080351

**Published:** 2024-08-04

**Authors:** Mingrui Zhang, Yingyue Cui, Xiaoying Mei, Longxian Li, Haotian Wang, Yingying Li, Yi Wu

**Affiliations:** 1 State Key Laboratory of Animal Nutrition and Feeding, College of Animal Science and Technology, China Agricultural University, Beijing 100193, China; zhangmingrui@cau.edu.cn (M.Z.); sy20233040846@cau.edu.cn (Y.C.);; 2 Hangzhou Wangmiao Biotechnology Co., Ltd., Hangzhou 311113, China

**Keywords:** probiotic, oral microbiota, cat, gingiva, tooth surfaces, tongue

## Abstract

**Simple Summary:**

Domestic cats kept in modern household environments are susceptible to various oral health issues, including halitosis, tartar accumulation, periodontal disease, and gingivitis. While these issues may initially go unnoticed, they can become challenging to manage as they progress. Proactive measures akin to human oral care, such as tooth brushing and dietary modifications, are crucial for preventing oral diseases in cats. Probiotics are widely employed in the prevention and treatment of oral diseases in humans, providing pet owners with a viable long-term preventive approach compared to brushing. Within this context, our study found that supplementing a composite probiotic formulation can inhibit potential pathogens in the oral cavity of cats and promote the growth of beneficial or commensal bacteria, potentially enhancing oral health in cats.

**Abstract:**

Probiotics demonstrated effectiveness in modulating oral microbiota and improving oral health in humans and rodents. However, its effects and applications on the oral microbiota of cats remain underexplored. Twelve healthy cats were randomly assigned to a control group (CON) and a composite probiotic group (CPG) for a 42-day trial. The CPG diet included additional supplementation of *Bifidobacterium animalis* subsp. *lactis* HN019, *Lactobacillus acidophilus* NCFM, and *Lactobacillus casei* LC-11, each at approximately 1 × 10^10^ CFU/kg. On days 0 and 42, microbial samples were collected from the gingiva, tooth surfaces, and tongue of all cats for 16S rRNA gene sequencing. Bacteroidetes, Firmicutes, and Proteobacteria were the dominant phyla across all oral sites. The CPG treatment enriched seven genera, such as *Moraxella*, *Actinomyces*, and *Frederiksenia* in the gingiva. Meanwhile, *Bergeyella* and *Streptococcus* were enriched on the tooth surfaces, while *Bergeyella*, *Flavobacterium*, and *Luteimonas* were enriched on the tongue. Furthermore, the composite probiotic effectively suppressed eight genera, such as *Bacteroides*, *Desulfovibrio*, and *Filifactor* in the gingiva of CPG cats, as well as *Helcococcus, Lentimicrobium,* and *Campylobacter* on tooth surfaces, and *Porphyromonas, Treponema,* and *Fusibacter* on the tongue. These findings suggest that the composite probiotic used in this study modulates the feline oral microbiota by supporting beneficial or commensal bacteria and inhibiting oral pathogens, demonstrating potential to improve oral health in cats.

## 1. Introduction

Oral health plays a crucial role in animal well-being, serving as the primary gateway for immune and digestive systems. In modern household management, cats are frequently diagnosed with oral health issues such as halitosis, dental calculus, and periodontal disease [1]. Mild oral problems such as halitosis can impact emotional bonding between cats and their owners, while severe conditions such as gingivitis and periodontitis lead to inflammation and pain in cats’ mouths, affecting their ability to eat and overall health [2]. The oral microbiota, the second-largest microbial reservoir in animals after the gut, is closely linked to the occurrence and progression of feline oral diseases [3,4]. Microorganisms play a pivotal role in the formation of dental plaque, which can trigger serious oral diseases, including periodontitis and gingivitis [5]. Typically, dysbiosis of the oral microbiota promotes proliferation and colonization of microorganisms within biofilms, thereby facilitating the formation of dental plaque and persistent dental calculus [3,6]. Moreover, if dental calculus forms above and below the gum line, the accumulation of dental plaque and its metabolites can irritate and induce tissue inflammation, leading to gingivitis and periodontitis [7].

Once dental calculus forms, professional periodontal treatment such as scaling is necessary to remove oral biofilm [8]. Therefore, preventive measures against dental plaque and calculus formation through daily oral care are crucial. However, maintaining regular physical brushing or chemical adjunct rinsing for cats may pose a challenge for owners. Hence, dietary interventions to modulate the balance of oral microbiota on a daily basis might offer a long-term effective approach for preventing oral issues in household cats. Probiotics may influence oral microbiota through mechanisms such as competitive inhibition, antimicrobial substance production, and immunoregulation [9,10], proving to be a promising strategy in alleviating periodontal diseases in humans and rodents [11,12]. Probiotics, including *Bifidobacterium animalis* subsp. *lactis* HN019 [11], *Lactobacillus acidophilus* NCFM [13], and *Lactobacillus casei* LC-11 [14] were demonstrated to improve the oral environment by inhibiting pathogenic strains’ growth, creating an acidic environment, and suppressing biofilm formation. Therefore, supplementing composite probiotics in diets may have potential benefits for promoting oral health in cats. However, current research on the effects of probiotics on cats primarily focused on gastrointestinal health, leaving a gap in understanding their impact on feline oral microbiota.

The oral cavity exhibits a complex structure, where varied microbial characteristics are determined by the growth conditions of different sites [15]. Generally, aerobic microorganisms tend to colonize areas more exposed to air, such as the tongue, oral mucosa, and tooth surfaces, while gingival crevices and subgingival areas favor anaerobic bacterial growth [16]. A study indicated that seven distinct sites within the human oral cavity harbor three types of bacterial communities, with significant differences observed between the microbial communities in subgingival and supragingival regions compared to other sites (hard palate, keratinized gingiva, buccal mucosa, tongue, and saliva) [17]. In addition, comparable research was conducted with dogs. A series of studies demonstrated that the oral microbial community of dogs exhibits different diversity and composition influenced by factors such as habitat surface type, pH, and oxygen tension [18,19]. Moreover, bacterial communities in supragingival and subgingival sites are most closely associated with oral diseases in dogs [20]. However, previous studies on the sequencing of feline oral microbiota mainly relied on samples collected from a single site or a mixture of multiple sites, with little focus on the simultaneous assessment of microbial variations across different sites within the feline oral cavity in a single study.

Therefore, this study supplemented three probiotic strains known for their beneficial effects on oral health in humans and rodents into the daily diet of cats. Utilizing high-throughput sequencing techniques, we analyzed the microbial composition changes across three oral sites (gingiva, tooth surfaces, and tongue) in cats to evaluate the potential effects of oral administration of the composite probiotic on modulating microbial compositions and improving the oral environment. This research aims to support the maintenance of feline oral microbiota balance and proposes novel nutritional strategies for promoting oral health in cats.

## 2. Materials and Methods

### 2.1. Animals

The study was approved by the Institutional Animal Care and Use Committee of China Agricultural University (AW50503202-2-6). Twelve neutered cats, consisting of six Chinese domestic cats and six British Shorthair cats, with an equal distribution of males and females, were recruited for the study. These cats had not received any oral care previously, but were found to be free from any oral diseases. Before the start of the research, a medical examination was performed on the experimental cats, which included blood and serum analyses, appetite, body condition, fecal score, and parasites. The results indicate that all cats were in good health. During the three months preceding the experiment, the cats were not administered antibiotics, therapeutic drugs, or any diets targeting the studied function. The cats had a median weight of 4.35 kg (with a range of 3.62–5.15 kg) and a median age of 3 years (with a range of 2–4 years). Based on a 9-point body condition score scale, these cats had a similar body condition score of 5.50 ± 0.50 points. All cats were housed at the Pet Feeding Center, Ministry of Agriculture and Rural Affairs Feed Industry Centre, China Agricultural University, and received care in accordance with the National Research Council’s guidelines.

### 2.2. Study Design

This study randomly assigned twelve cats into two groups, with six cats in each group evenly distributed by gender and breed. The control group (CON) was fed a basal diet, while the composite probiotic group (CPG) received the basal diet with a post-sprayed composite probiotic formulation. The CPG diet contained *Bifidobacterium animalis* subsp. *lactis* HN019, *Lactobacillus acidophilus* NCFM, and *Lactobacillus casei* LC-11, each at approximately 1 × 10^10^ CFU/kg. The composite probiotic preparation was purchased from Aikoyou Health Technology Co., Ltd. (Suzhou, China). The probiotic supplement is a powder formulated with maltodextrin as a carrier and a mixture of *Bifidobacterium animalis* subsp. *lactis* HN019, *Lactobacillus acidophilus* NCFM, and *Lactobacillus casei* LC-11, each at a concentration of approximately 2 × 10^10^ CFU/g. The experimental diets for cats in both CON and CPG groups were prepared by Hangzhou Wangmiao Biotechnology Co., Ltd. (Hangzhou, China). The nutritional level and composition of the basal diet are presented in Table 1. According to the Official Methods of Analysis of AOAC International [21], feed ingredients were analyzed in terms of moisture, crude protein, crude fat, crude fiber, and ash. All chemical compositions were analyzed in duplicate. The study lasted for 42 days, during which each cat had *ad libitum* access to food and water. Before the formal trial, all cats were fed with the same basal diet for 30 days. The feeding rate and all nutritional requirements met the NRC (2006) recommendations for adult cats.

### 2.3. Sample Collection

On days 0 and 42 of the experiment, microbial samples were collected from the gums, tooth surfaces, and tongue of all cats. To minimize food residue in the oral cavity, the cats’ diet was removed 8 h prior to sampling, while water was freely available. All procedures were conducted using sterile gloves and standard aseptic techniques. Sterile swabs were gently brushed over the upper and lower gums, as well as the dorsal and ventral surfaces of the tongue to collect microbial samples, avoiding contact with the teeth. Each swab was wiped three times on each side, for 5 s each time. Tooth surface microbes were collected using sterile swabs from teeth labeled as 104, 204, 108, and 208 in the upper jaw and 304, 404, 309, and 409 in the lower jaw, according to the modified Triadan system [22]. Three swab samples were taken from each site of each cat, individually placed in cryotubes, and immediately stored at −80 °C.

### 2.4. Oral Microbiota Analysis

Total microbial genomic DNA was extracted from the swab samples using the E.Z.N.A.^®^ soil DNA kit (Omega Bio-tek, Norcross, GA, USA). Amplification of 16S rRNA from the V3-V4 regions was performed using primer 338F (5′-ACTCCTACGGGAGGCAGCAG-3′) and primer 806R (5′-GGACTACHVGGGTWTCTAAT-3′). The purified PCR products were combined in equimolar proportions and subjected to paired-end sequencing using the Illumina MiSeq platform (San Diego, CA, USA). The demultiplexed sequencing reads were subjected to quality control using fastp software (v0.19.6) and spliced with Flash software (v1.2.11) following the method described in our previous study [23]. Subsequently, the feature table containing operational taxonomic units (OTUs) was generated employing UPARSE (v11) with a 97% similarity. To annotate the sequences of OTUs, the RDP classifier Bayesian algorithm (v2.13), with a confidence threshold of 0.7, was applied based on the Silva138/16S_bacteria database. The community composition of each sample was analyzed at different taxonomic levels. Mothur (v1.30.2) was utilized to assess the α-diversity via Faith’s phylogenetic diversity (Faith’s PD) as well as Ace, Chao, Simpson, Shannon, and Sobs indexes. β-diversity was evaluated by calculating the weighted UniFrac distance with ANOSIM examination and then visualized through principal coordinate analysis (PCoA). Linear discriminant analysis effect size (LEfSe) was employed to identify distinct differences in bacterial taxa at the phylum, family, and genus levels between the two treatments (LDA score > 3 or 4, *p* < 0.05).

### 2.5. Statistical Analysis

Statistical analysis was conducted on all data using IBM SPSS 26.0 (Chicago, IL, USA), and graphical representation of α-diversity was generated using GraphPad Prism 9.0 (San Diego, CA, USA). The raw sequencing data of microbiomes were analyzed using R tools to generate visualizations. Bar plots for the microbiota composition were created using the R ggplot package, and community heatmap analysis was generated using the R vegan package. The Wilcoxon rank-sum test was applied to assess intergroup differences in diversity indices and microbiota abundance. Multiple testing correction was performed using the false discovery rate method. Bootstrap resampling was employed to calculate the confidence intervals with a 95% confidence level. *p* < 0.05 was deemed statistically significant. The results are presented as the means ± SEMs.

## 3. Results

### 3.1. Oral Microbial Composition in Cats at Baseline

**Figure 1:**
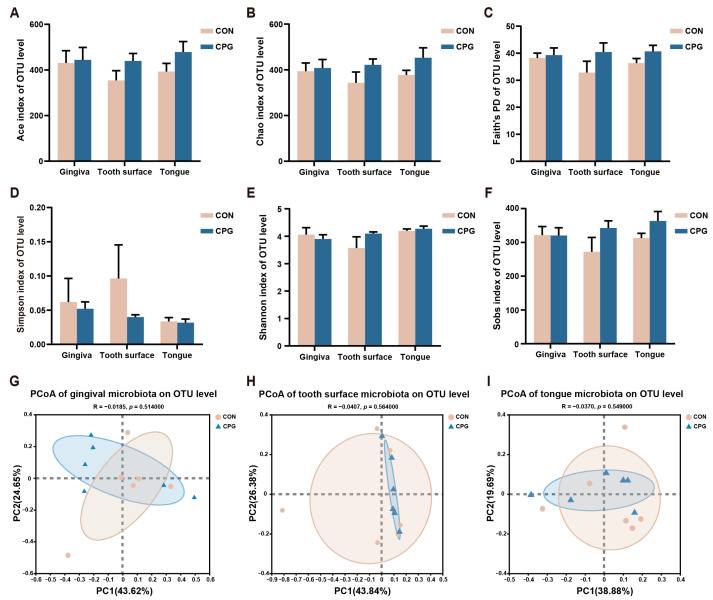
Differences in α- and β-diversity of oral microbiota in cats at baseline. (**A**–**F**) Ace index, Chao index, Faith’s phylogenetic diversity, Simpson index, Shannon index, and Sobs index at the OTU level. (**G**–**I**) Principal coordinate analysis (PCoA) based on weighted UniFrac distance at the OTU level of cat gum, tooth surface, and tongue microbiota. CON, cats fed with a basal diet; and CPG, cats received the basal diet with a post-sprayed composite probiotic formulation. Values are mean ± SEM (*n* = 6).

Illumina sequencing yielded an average of 58,846 16S rRNA amplicon sequences per sample post quality filtering. Subsequent analyses were performed on all samples rarified to a depth of 18,583 sequences. The α-diversity indicators comprised Ace, Chao, Faith’s PD, Simpson, Shannon, and Sobs. At the experiment’s baseline, no significant differences in microbial α-diversity were observed between the CON and CPG groups on the gingiva, tooth surfaces, and tongue (*p* ≥ 0.05, Figure 1A–F). Furthermore, employing weighted-UniFrac distances, PCoA analysis demonstrated no significant separation among the microbiota communities of the gingiva, tooth surfaces, and tongue in the CON and CPG groups at the baseline (*p* ≥ 0.05, Figure 1G–I). The relative abundance of feline oral microbiota with distinct differences at the phylum, family, and genus levels is shown in Table 2. In the gingival region, the abundance of Desulfomicrobiaceae, Caulobacteraceae, *Desulfomicrobium,* and *norank_f_Propionibacteriaceae* was significantly increased in the CPG group compared with the CON group (*p* < 0.05). Additionally, the relative abundance of *Parabacteroides*, *Granulicatella,* and *unclassified_f_Anaerovoracaceae* was lower in the CPG group than that in the CON group (*p* < 0.05). On the tooth surfaces, a significant increase in the relative abundance of Campilobacterota, unclassified_c_Gammaproteobacteria, *Frederiksenia*, *unclassified_c_Gammaproteobacteria,* and *norank_f_Pasteurellaceae* was observed in the CPG group compared to the CON group (*p* < 0.05). At the tongue site, the abundance of Synergistota, Synergistaceae, *Fretibacterium,* and *Prevotellaceae_UCG-003* was markedly decreased in the CPG group compared with the CON group (*p* < 0.05). Moreover, the abundance of *unclassified_f_Lachnospiraceae* was higher in the CPG group than that in the CON group (*p* < 0.05).

### 3.2. Effects of Composite Probiotics on α- and β-Diversity of Oral Microbiota in Cats

**Figure 2:**
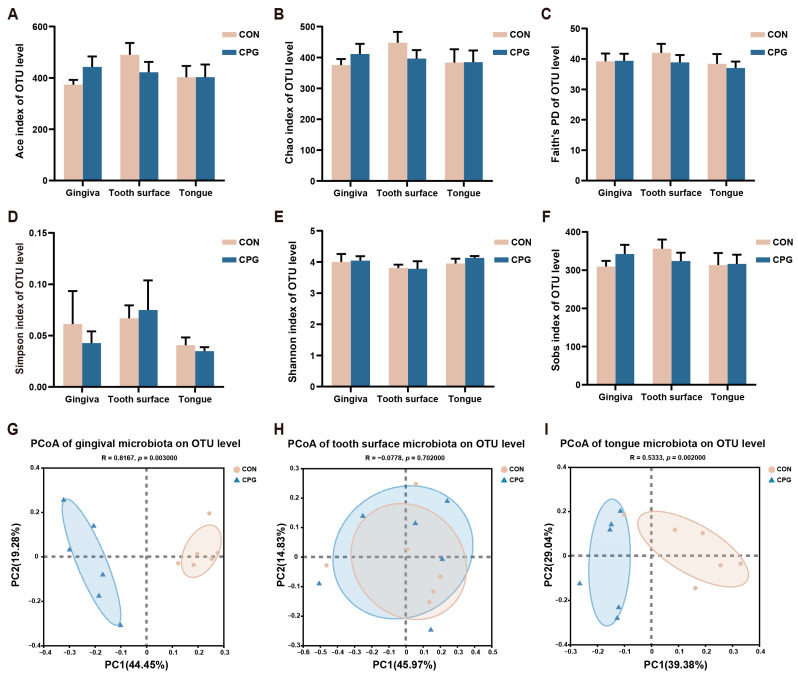
Differences in α- and β-diversity of oral microbiota in different parts of cats on day 42. (**A**–**F**) Ace index, Chao index, Faith’s phylogenetic diversity, Simpson index, Shannon index, and Sobs index at the OTU level. (**G**–**I**) Principal coordinate analysis (PCoA) based on weighted UniFrac distance at the OTU level of cat gum, tooth surface, and tongue microbiota. CON, cats fed with a basal diet; and CPG, cats received the basal diet with a post-sprayed composite probiotic formulation. Values are mean ± SEM (*n* = 6).

The oral microbial diversity of cats in different treatments is illustrated in Figure 2. On day 42, the CON and CPG treatments did not exhibit any significant differences in terms of microbial community diversity (Faith’s PD, Simpson, and Shannon), nor in community richness (Ace, Chao, and Sobs indexes), across the gingival, tooth surface, and tongue regions of cats (*p* ≥ 0.05, Figure 2A–F). However, the PCoA result using weighted-UniFrac distances reveals a discernible segregation of microbial communities between the two experimental treatments, with distinct clustering observed in both the gingival and tongue samples (*p* < 0.05, Figure 2G,I). In addition, no significant clustering of microbial communities on the tooth surfaces was observed between the CON and CPG groups in the weighted PCoA plots (*p* ≥ 0.05, Figure 2H).

### 3.3. Effects of Composite Probiotics on the Composition of Gingival Microbiota in Cats

**Figure 3:**
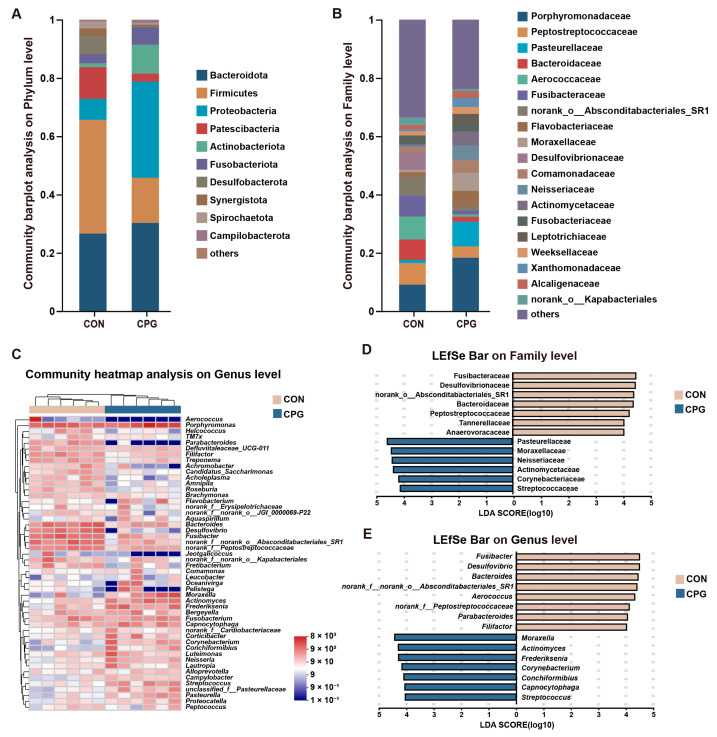
Effects of different treatments on the composition of gingival microbiota in cats on day 42. (**A,B**) Community barplot analysis on the phylum and family levels. (**C**) Community heatmap analysis on the genus level. (**D,E**) Linear discriminant analysis effect size (LEfSe) analysis on the family and genus levels. CON, cats fed with a basal diet; and CPG, cats received the basal diet with a post-sprayed composite probiotic formulation.

For gingival microbiota in cats, a total of 407 shared OTUs were identified, alongside 173 (CON) and 427 (CPG) specific OTUs. The gingival microbiota was categorized into 17 phyla, 34 classes, 83 orders, 143 families, 255 genera, and 480 species. The top 3 of the gingival microbial abundance in each group at the phylum level were both: Bacteroidota (CON: 26.59%; CPG: 30.27%), Firmicutes (CON: 39.07%; CPG: 15.50%), and Proteobacteria (CON: 7.18%; CPG: 32.97%), comprising over 70% of the total sequences (Figure 3A). Furthermore, the abundance of Firmicutes, Patescibacteria, Desulfobacterota and Spirochaetota was markedly reduced in the CPG treatment compared with those in the CON treatment (*p* < 0.05, Table 3). The abundances of Proteobacteria and Actinobacteriota were higher in the CPG treatment than those in the CON treatment (*p* < 0.05, Table 3).

The most abundant families of gingival microbiota (Figure 3B) included Porphyromonadaceae (CON: 9.08%; CPG: 18.32%), Peptostreptococcaceae (CON: 7.41%; CPG: 3.93%), Pasteurellaceae (CON: 1.11%; CPG: 8.41%), and Bacteroidaceae (CON: 6.97%; CPG: 1.73%). In terms of genus (Figure 3C), the dominant bacteria were *Porphyromonas* (CON: 9.08%; CPG: 18.32%), *Bacteroides* (CON: 6.97%; CPG: 1.73%), and *Fusibacter* (CON: 7.24%; CPG: 1.37%). Further, using the LEfSe (LDA score > 4) in conjunction with the Wilcoxon rank-sum test for differential analysis, our results indicate significant enrichment of Fusibacteraceae, Desulfovibrionaceae, norank_o_Absconditabacteriales_SR1, Bacteroidaceae, Peptostreptococcaceae, Anaerovoracaceae, Tannerellaceae, *Fusibacter*, *Desulfovibrio*, *Bacteroides*, *norank_f_norank_o_Absconditabacteriales_SR1*, *Aerococcus*, *norank_f_Peptostreptococcaceae*, *Parabacteroides,* and *Filifactor* in the gingival samples of the CON group (*p* < 0.05), while Pasteurellaceae, Moraxellaceae, Neisseriaceae, Actinomycetaceae, Corynebacteriaceae, Streptococcaceae, *Moraxella*, *Actinomyces*, *Frederiksenia*, *Capnocytophaga*, *Corynebacterium*, *Conchiformibius,* and *Streptococcus* were notably enriched in the gingival microbiota of the CPG group (*p* < 0.05, Figure 3D,E and Table 3).

### 3.4. Effects of Composite Probiotics on the Composition of Tooth Surface Microbiota in Cats

**Figure 4:**
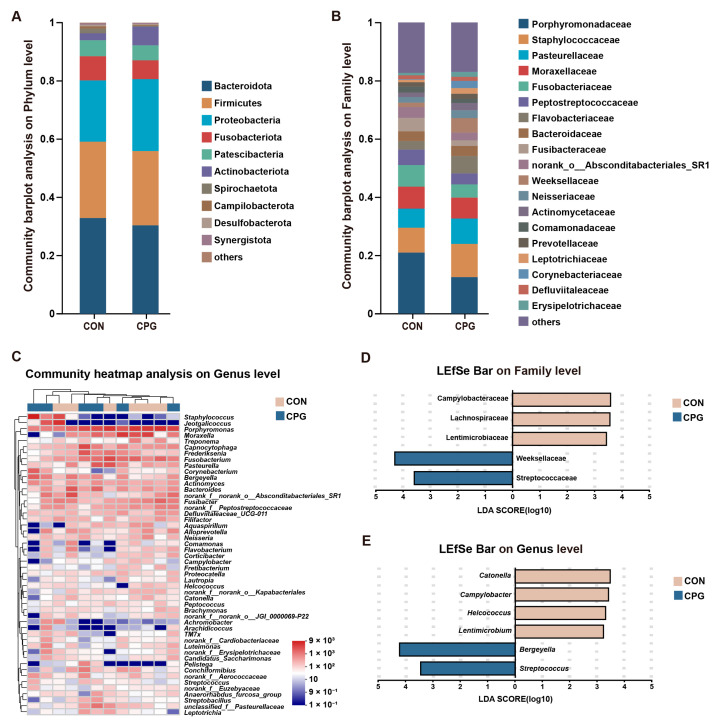
Effects of different treatments on the composition of tooth surface microbiota in cats on day 42. (**A,B**) Community barplot analysis on the phylum and family levels. (**C**) Community heatmap analysis on the genus level. (**D,E**) Linear discriminant analysis effect size (LEfSe) analysis on the family and genus levels. CON, cats fed with a basal diet; and CPG, cats received the basal diet with a post-sprayed composite probiotic formulation.

For tooth surface microbiota in cats, a total of 524 shared OTUs were identified, alongside 360 (CON) and 308 (CPG) specific OTUs. The taxonomic classification of the microbiota encompassed 17 phyla, 32 classes, 74 orders, 129 families, 233 genera, and 445 species. In terms of phylum (Figure 4A), the dominant bacteria were Bacteroidota (CON: 32.90%; CPG: 30.43%), Firmicutes (CON: 26.23%; CPG: 25.52%), and Proteobacteria (CON: 21.06%; CPG: 24.67%), accounting for approximately 80% of all bacterial phyla. In terms of family (Figure 4B), the Porphyromonadaceae (CON: 20.97%; CPG: 12.54%), Staphylococcaceae (CON: 8.52%; CPG: 11.41%), Pasteurellaceae (CON: 6.55%; CPG: 8.69%), and Moraxellaceae (CON: 7.56%; CPG: 7.19%) were dominated. In addition, the most abundant genera of tooth surface bacteria (Figure 4C) included *Porphyromonas* (CON: 20.97%; CPG: 12.54%), *Moraxella* (CON: 7.56%; CPG: 7.19%), and *Staphylococcus* (CON: 2.94%; CPG: 9.23%). Although PCoA results indicate no significant impact of dietary supplementation with the composite probiotic on the bacterial structure of cat tooth surfaces, the LEfSe analysis (LDA score > 3) identified enrichment of five families and six genera on the tooth surfaces of both treatments. In detail, the CPG treatments exhibited a significantly elevated abundance of Weeksellaceae, Streptococcaceae, *Bergeyella*, and *Streptococcus* compared to the CON treatments (*p* < 0.05, Figure 4D,E and Table 4). Meanwhile, dietary supplementation with the composite probiotic markedly decreased the abundance of Lachnospiraceae, Campylobacteraceae, Lentimicrobiaceae, *Catonella*, *Helcococcus*, *Campylobacter,* and *Lentimicrobium* on the tooth surfaces of cats compared with the CON treatment (*p* < 0.05, Figure 4D,E and Table 4).

### 3.5. Effects of Composite Probiotics on the Composition of Tongue Microbiota in Cats

**Figure 5:**
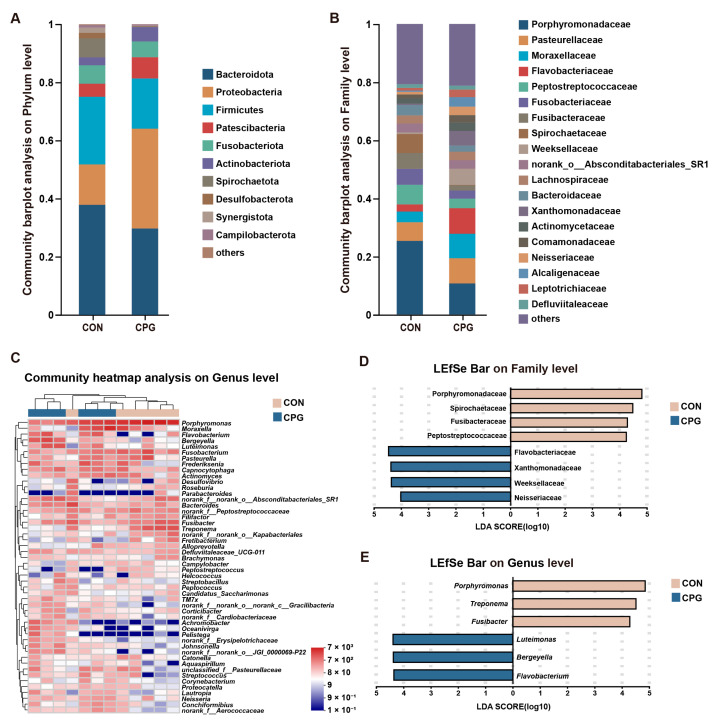
Effects of different treatments on the composition of tongue microbiota in cats on day 42. (**A,B**) Community barplot analysis on the phylum and family levels. (**C**) Community heatmap analysis on the genus level. (**D,E**) Linear discriminant analysis effect size (LEfSe) analysis on the family and genus levels. CON, cats fed with a basal diet; and CPG, cats received the basal diet with a post-sprayed composite probiotic formulation.

For tongue microbiota in cats, a total of 463 shared OTUs were identified, alongside 342 (CON) and 259 (CPG) specific OTUs. The tongue microbiota was categorized into 18 phyla, 35 classes, 81 orders, 139 families, 246 genera, and 449 species. In terms of phylum (Figure 5A), the Bacteroidota (CON: 37.87%; CPG: 29.72%), Proteobacteria (CON: 13.90%; CPG: 34.36%), and Firmicutes (CON: 23.28%; CPG: 17.26%) were dominated. The results of the Wilcoxon rank-sum test indicate a distinct increase in the abundance of Proteobacteria (*p* < 0.05) and a significant decrease in the abundance of Spirochaetota (*p* < 0.05) in the tongue microbiota of the CPG group compared to the CON group (Table 5). At the family level (Figure 5B), the most abundant bacteria were Porphyromonadaceae (CON: 25.33%; CPG: 10.67%), Pasteurellaceae (CON: 6.42%; CPG: 8.70%), Moraxellaceae (CON: 3.67%; CPG: 8.41%), and Flavobacteriaceae (CON: 2.46%; CPG: 8.79%). The dominant genera (Figure 5C) included *Porphyromonas* (CON: 25.33%; CPG: 10.67%), *Moraxella* (CON: 3.66%; CPG: 8.40%), and *Fusobacterium* (CON: 5.46%; CPG: 2.82%). LEfSe analysis (LDA score > 4) identified enrichment of eight families and six genera in the two treatments. Among them, four families (Porphyromonadaceae, Spirochaetaceae, Fusibacteraceae, and Peptostreptococcaceae) were enriched in the CON group (*p* < 0.05), while four families (Flavobacteriaceae, Xanthomonadaceae, Weeksellaceae, and Neisseriaceae) were enriched in the CPG group (*p* < 0.05, Figure 5D and Table 5). Additionally, three genera (*Porphyromonas*, *Treponema,* and *Fusibacter*) were significantly enriched in the CON group (*p* < 0.05), while three genera (*Luteimonas*, *Bergeyella,* and *Flavobacterium*) were markedly enriched in the CPG group (*p* < 0.05, Figure 5E and Table 5).

## 4. Discussion

The oral cavity harbors a diverse microbiota that plays a crucial role in the occurrence and progression of oral diseases [24]. Dietary interventions aimed at modulating the structure and composition of the oral microbiota may offer approaches for improving oral health. Studies indicated that supplementing oral probiotics in daily diets can regulate the oral microbiome, effectively alleviating oral issues in humans and rodents [11,12]. However, there is currently a lack of research documenting the effects of oral probiotics on the oral microbiota of cats. Therefore, this study selected a composite probiotic preparation to investigate its regulatory effects on the oral microbiota of cats. The current results suggest that supplementing with the composite probiotic may alter the composition of the oral microbiota in different oral niches of cats. This is primarily manifested by an increase in the abundance of potentially beneficial or commensal bacteria such as *Moraxella*, *Actinomyces*, and *Frederiksenia* in the gingiva, *Bergeyella,* and *Streptococcus* on tooth surfaces, and *Bergeyella*, *Flavobacterium*, and *Luteimonas* on the tongue. Additionally, there is a decrease in the abundance of taxa associated with oral diseases, including *Bacteroides*, *Desulfovibrio*, and *Filifactor* in the gingiva, *Helcococcus* and *Campylobacter* on tooth surfaces, and *Porphyromonas* and *Treponema* on the tongue.

This study utilized a composite probiotic preparation composed of *Bifidobacterium animalis* subsp. *lactis* HN019, *Lactobacillus acidophilus* NCFM, and *Lactobacillus casei* LC-11. A previous in vitro study demonstrated that *Bifidobacterium* can inhibit the growth of periodontal pathogens, particularly *Porphyromonas gingivalis*, without inhibiting the beneficial oral bacterium *Streptococcus* [25]. Additionally, a human clinical study indicated that *Bifidobacterium animalis* subsp. *lactis* HN019 alleviated systemic inflammation in patients with advanced chronic periodontitis, reduced periodontal pathogen colonization, and potentially colonized subgingival biofilms [12]. The beneficial effects of *Lactobacillus* were extensively documented, including anti-inflammatory properties [26], reduction in halitosis [27], inhibition of periodontal pathogens [28], and decrease in biofilm formation through its metabolites, such as organic acids, hydrogen peroxide, and bacteriocins [29,30]. Furthermore, studies indicated that *Lactobacillus acidophilus* and *Lactobacillus casei* can ameliorate periodontal inflammation and modulate oral microbiota composition [13,14,31]. This study selected 12 healthy adult cats for a 42-day randomized controlled trial. Considering the individual variations among cats, we also compared the differences in oral microbial composition between the two groups at baseline. Baseline results indicate no significant differences in the α- and β-diversity of microbial communities on the gingiva, tooth surfaces, and tongue between the two groups before the start of the trial. Studies suggested that cats with gingivostomatitis and periodontitis tend to exhibit higher bacterial α-diversity indices (Shannon and Chao1) compared to healthy cats [3], although differences are not always observed [32]. Moreover, oral diseases in humans are associated with increased microbial diversity and abundance [33]. In this study, supplementation with the composite probiotic for 42 days did not result in significant changes in bacterial diversity and richness on the gingiva, tooth surfaces, and tongue, which is considered reasonable in the oral cavity of healthy cats. Furthermore, previous research demonstrated that diseased cats exhibit microbial dysbiosis in the oral cavity, as evidenced by β-diversity results showing significant separation of bacterial communities from healthy cats [3]. Dietary interventions can modulate the microbial structure and composition in the oral cavity of cats [34], and notably, by day 42 of the trial, supplementation with the composite probiotic preparation significantly altered the bacterial structure on the gingiva and tongue.

Further analysis of the oral microbiota composition revealed a consistent dominance of the bacterial phyla Bacteroidota, Firmicutes, and Proteobacteria, whether on the cat’s gums, the tooth surfaces, or tongues, aligning with the microbial composition observed in the oral cavity of healthy cats [35]. Previous reports indicated higher abundance of bacterial phyla such as Bacteroidota, Firmicutes, Synergistota, Chloroflexi, Fusobacteria, and Spirochaetota in cats suffering from periodontitis and gingivostomatitis compared to healthy counterparts, whereas Proteobacteria are more abundant in the oral cavity of healthy cats [3,36]. In this study, supplementation with probiotics resulted in a decrease in Spirochaetota on the gingiva and tongue of cats, accompanied by an increase in Proteobacteria, while Firmicutes decreased on the gingiva. Further, a significant reduction in *Treponema*, which is considered a marker of periodontal disease, was observed on the tongue. *Treponema*, belonging to the phylum Spirochaetota, amplifies the inflammatory response of host normal tissues by metabolizing cytotoxic products and utilizing its own structure and specific adhesion [37]. Moreover, Actinobacteriota, serving as the initial colonizers of the oral cavity, are compatible with periodontal health and ecological succession [36,38]. Actinobacteriota, particularly *Actinomyces* and *Corynebacterium*, exhibited significant enrichment at the gingiva of healthy cats supplemented with probiotics, which is consistent with previous studies [3,4]. *Desulfovibrio*, belonging to the phylum Desulfobacterota, was identified as a potential biomarker of periodontitis, with a significant decrease in abundance observed at the gingiva after probiotic supplementation in cats [39].

In this study, both Porphyromonadaceae and Pasteurellaceae consistently ranked among the top three most abundant bacterial families in the three oral sites of cats. Indeed, Porphyromonadaceae and Pasteurellaceae were described as the most prevalent bacterial taxa in the oral cavity of healthy cats [35]. Notably, *Porphyromonas*, although among the most abundant genera in the oral cavity of cats, is highly correlated with the prevalence of periodontal disease [40]. Some of these pathogenic bacteria can selectively inhibit bactericidal activity by evading host immune responses, thereby leading to microbiota dysbiosis and inflammatory microenvironments [41,42]. Additionally, *Porphyromonas* secretes lipopolysaccharides and extracellular proteases that degrade the soft tissues surrounding the teeth [42]. Conversely, studies demonstrated that the relative abundance of *Frederiksenia*, a genus within the Pasteurellaceae family, is increased in multiple oral sites of healthy cats compared to cats with gingivitis [4]. Our study found a decrease in *Porphyromonas* on the tongue and an increase in *Frederiksenia* on the gingiva of cats following supplementation with a composite probiotic, suggesting a potential protective effect on feline oral health. Furthermore, clinical research showed that, in addition to *Porphyromonas*, *Moraxella* and *Fusobacterium* are also dominant genera in the oral cavity of healthy cats [3], consistent with our findings. Interestingly, we observed differences in the abundance and order of dominant bacteria at the phylum, family, and genus levels across the gingiva, tooth surfaces, and tongue. Oral bacteria exhibit high variability, and microbial communities in different habitats may vary due to differences in host oral pH, oxygen supply, cell types, mucosal characteristics, and immune factors [17,43]. Additionally, it is noteworthy that microbial communities in the gingiva, tooth surfaces, and tongue exhibited different responses to probiotic supplementation, suggesting specific effects of the composite probiotic on the oral microbiota.

This study also found that supplementation with a composite probiotic for 42 days resulted in increased abundance of *Streptococcus* on the gingiva and tooth surfaces of cats, along with an elevated abundance of *Bergeyella* on the tongue and tooth surfaces. Previous research indicated that the abundance of *Streptococcus* and *Bergeyella*, as oral symbiotic bacteria, is negatively correlated with the development of dental calculus, plaque, and oral diseases [19,35]. Moreover, it was theorized in previous studies that the subgingival area is anaerobic and rich in peptides, and changes in the oral environment may promote overgrowth of Gram-negative and anaerobic bacteria, thereby driving the progression of oral diseases [44,45]. Consequently, cats with periodontal and gingival diseases showed a significant increase in Gram-negative and anaerobic bacteria, as reported in previous studies [3,32]. In addition to the bacteria mentioned above, this study also observed a significant decrease in the abundance of Gram-negative bacteria such as *Bacteroides* and *Parabacteroides* at the gingival, and *Campylobacter* and *Lentimicrobium* on the tooth surfaces in cats orally administered probiotics. Meanwhile, the CPG group also exhibited a reduction in several bacterial genera previously associated with oral diseases, including *Filifactor* [3], *Aerococcus* [46], and *Helcococcus* [47], and an increase in beneficial or commensal bacteria of healthy cats in the subgingival and tongue regions, including *Capnocytophaga* [48], *Conchiformibius* [4], *Flavobacterium* [4], and *Luteimonas* [49]. These findings suggest that the composite probiotic used in this study may potentially offer protective effects on feline oral health by balancing the oral microbiota and inhibiting the proliferation of bacteria associated with periodontal diseases.

This study has certain limitations. Firstly, the experimental period lasted for 42 days, and to observe more significant effects, longer-term feeding studies may be necessary. Secondly, this study focused on relatively small sample sizes of orally healthy cats, aiming to provide support for the use of composite probiotics in preventing oral diseases. Further research should involve larger cohorts of cats with existing oral diseases and explore the therapeutic effects of composite probiotics. Additionally, while this study offers valuable insights into the impact of probiotics on the oral microbiota of cats, further research is needed to elucidate the underlying mechanisms driving these observed changes, thus providing a more comprehensive understanding of the actions and applications of composite probiotics.

## 5. Conclusions

In this study, supplementation of the composite probiotic (*Bifidobacterium animalis* subsp. *lactis* HN019, *Lactobacillus acidophilus* NCFM, and *Lactobacillus casei* LC-11) was associated with changes in the oral microbiota of healthy cats. Microbial communities at different oral sampling sites responded differently to the probiotic formulation, with a decrease in potential pathogen abundance observed, including *Bacteroides*, *Desulfovibrio*, and *Filifactor* in the gingiva, *Helcococcus* and *Campylobacter* on tooth surfaces, and *Porphyromonas* and *Treponema* on the tongue. Additionally, the abundance of commensal or beneficial microbiota increased in the cat’s oral cavity after probiotic supplementation, such as *Moraxella*, *Actinomyces*, and *Frederiksenia* in the gingiva, *Bergeyella* and *Streptococcus* on tooth surfaces, and *Bergeyella*, *Flavobacterium*, and *Luteimonas* on the tongue. Therefore, the composite probiotic studied may contribute to promoting oral health in cats, but further research is needed to validate its potential for preventing or assisting in the treatment of oral diseases.

## Figures and Tables

**Table 1:** Dietary composition and nutritional level of basal diet.

Diet Composition	%	Nutrient Content	%
Chicken meal	54.50	Moisture	7.12
Chicken fat	8.00	Crude protein	41.65
Fish oil	2.00	Crude fat	20.28
Tapioca	3.00	Crude fiber	1.82
Potato starch	19.00	Ash	7.87
Rice	4.00		
Chicken liver powder	5.00		
Alfalfa meal	3.00		
Choline chloride	0.30		
Salt	0.50		
Taurine	0.20		
Mineral complexes and vitamins ^1^	0.50		

^1^ Mineral complexes and vitamins provided the following per kilogram of feed: vitamin A (14,500 IU), vitamin D_3_ (1,000 IU), vitamin E (156 IU), and vitamin B_1_ (32.0 mg), vitamin B_2_ (30.0 mg), vitamin B_3_ (120 mg), vitamin B_5_ (88.0 mg), vitamin B_6_ (13.0 mg), vitamin B_12_ (0.20 mg); Fe (FeSO_4_) 100 mg, Cu (CuSO_4_) 7.00 mg, Co (CoSO_4_) 1.00 mg, I (CaI_2_) 20.0 mg, Mn (MnSO_4_) 20.0 mg, Zn (ZnSO_4_) 68.0 mg, and Se (Na_2_SeO_3_) 0.50 mg.

**Table 2:** Differences of oral microbial composition in cats at baseline.

Items	CON	CPG	*p*-Value
Gingiva			
Desulfomicrobiaceae	0.011 ± 0.004	0.096 ± 0.046	0.03
Caulobacteraceae	0	0.022 ± 0.013	0.03
*Parabacteroides*	1.743 ± 1.295	0.044 ± 0.028	0.03
*Granulicatella*	0.201 ± 0.156	0	0.01
*Desulfomicrobium*	0.011 ± 0.004	0.096 ± 0.046	0.03
*unclassified_f_Anaerovoracaceae*	0.071 ± 0.046	0	0.03
*norank_f_Propionibacteriaceae*	0.004 ± 0.004	0.031 ± 0.011	0.03
Tooth surface			
Campilobacterota	0.326 ± 0.284	0.839 ± 0.276	0.04
unclassified_c_Gammaproteobacteria	0.030 ± 0.015	0.209 ± 0.083	0.008
*Frederiksenia*	1.423 ± 0.644	4.214 ± 0.797	0.03
*unclassified_c_Gammaproteobacteria*	0.030 ± 0.015	0.209 ± 0.083	0.008
*norank_f_Pasteurellaceae*	0.004 ± 0.002	0.018 ± 0.005	0.03
Tongue			
Synergistota	0.770 ± 0.424	0.177 ± 0.058	0.04
Synergistaceae	0.770 ± 0.424	0.177 ± 0.058	0.04
*Fretibacterium*	0.767 ± 0.423	0.176 ± 0.059	0.04
*unclassified_f_Lachnospiraceae*	0.042 ± 0.018	0.145 ± 0.026	0.03
*Prevotellaceae_UCG-003*	0.079 ± 0.038	0.003 ± 0.003	0.004

CON, cats fed with a basal diet; and CPG, cats received the basal diet with a post-sprayed composite probiotic formulation. Values are mean ± SEM (*n* = 6). Statistical significance was determined at *p* < 0.05.

**Table 3:** Differential composition of gingival microbiota in cats on day 42.

Items	CON	CPG	*p*-Value
Firmicutes	39.074 ± 5.236	15.495 ± 2.310	0.005
Proteobacteria	7.181 ± 1.415	32.968 ± 6.508	0.008
Patescibacteria	10.917 ± 1.415	2.783 ± 0.804	0.005
Actinobacteriota	1.307 ± 0.252	9.877 ± 2.152	0.005
Desulfobacterota	6.355 ± 1.197	0.322 ± 0.112	0.005
Spirochaetota	2.344 ± 0.282	0.644 ± 0.230	0.008
Peptostreptococcaceae	7.414 ± 0.652	3.926 ± 0.886	0.02
Pasteurellaceae	1.107 ± 0.411	8.409 ± 1.961	0.005
Bacteroidaceae	6.970 ± 1.566	1.727 ± 0.511	0.02
Fusibacteraceae	7.240 ± 1.177	1.369 ± 0.549	0.005
norank_o_Absconditabacteriales_SR1	6.380 ± 1.326	1.222 ± 0.418	0.008
Moraxellaceae	0.822 ± 0.788	6.179 ± 2.563	0.03
Desulfovibrionaceae	6.192 ± 1.214	0.111 ± 0.081	0.005
Neisseriaceae	0.430 ± 0.148	5.002 ± 2.589	0.005
Actinomycetaceae	0.565 ± 0.156	4.832 ± 1.068	0.005
Corynebacteriaceae	0.218 ± 0.108	2.822 ± 1.379	0.008
Streptococcaceae	0.257 ± 0.090	2.764 ± 0.652	0.01
Anaerovoracaceae	2.690 ± 0.624	0.300 ± 0.101	0.005
Tannerellaceae	2.641 ± 0.496	0.330 ± 0.137	0.005
*Bacteroides*	6.970 ± 1.566	1.727 ± 0.511	0.02
*Fusibacter*	7.240 ± 1.177	1.369 ± 0.549	0.005
*Aerococcus*	7.636 ± 7.616	0	0.002
*norank_f_norank_o_* *Absconditabacteriales_SR1*	6.380 ± 1.326	1.222 ± 0.418	0.008
*Moraxella*	0.818 ± 0.788	6.086 ± 2.582	0.03
*Desulfovibrio*	6.187 ± 1.214	0.111 ± 0.081	0.005
*norank_f_Peptostreptococcaceae*	4.206 ± 0.521	1.558 ± 0.267	0.005
*Actinomyces*	0.565 ± 0.156	4.832 ± 1.068	0.005
*Frederiksenia*	0.688 ± 0.319	4.675 ± 1.473	0.02
*Capnocytophaga*	1.212 ± 0.406	3.532 ± 0.967	0.04
*Filifactor*	2.722 ± 0.540	0.719 ± 0.161	0.005
*Corynebacterium*	0.218 ± 0.108	2.822 ± 1.379	0.008
*Streptococcus*	0.257 ± 0.090	2.764 ± 0.652	0.01
*Parabacteroides*	2.362 ± 0.468	0.038 ± 0.038	0.004
*Conchiformibius*	0.057 ± 0.024	2.266 ± 1.236	0.005

CON, cats fed with a basal diet; CPG, cats received the basal diet with a post-sprayed composite probiotic formulation. Values are mean ± SEM (*n* = 6). Statistical significance was determined at *p* < 0.05.

**Table 4:** Differential composition of tooth surface microbiota in cats on day 42.

Items	CON	CPG	*p*-Value
Weeksellaceae	1.499 ± 0.822	4.919 ± 1.207	0.04
Lachnospiraceae	1.626 ± 0.182	0.904 ± 0.184	0.04
Streptococcaceae	0.170 ± 0.053	0.675 ± 0.220	0.04
Campylobacteraceae	0.600 ± 0.183	0.152 ± 0.090	0.04
Lentimicrobiaceae	0.037 ± 0.015	0.001 ± 0.001	0.02
*Bergeyella*	1.379 ± 0.837	4.612 ± 1.282	0.04
*Catonella*	0.887 ± 0.227	0.227 ± 0.057	0.02
*Helcococcus*	0.632 ± 0.138	0.259 ± 0.081	0.04
*Streptococcus*	0.167 ± 0.052	0.673 ± 0.221	0.04
*Campylobacter*	0.600 ± 0.183	0.152 ± 0.090	0.04
*Lentimicrobium*	0.037 ± 0.015	0.001 ± 0.001	0.02

CON, cats fed with a basal diet; CPG, cats received the basal diet with a post-sprayed composite probiotic formulation. Values are mean ± SEM (*n* = 6). Statistical significance was determined at *p* < 0.05.

**Table 5:** Differential composition of tongue microbiota in cats on day 42.

Items	CON	CPG	*p*-Value
Proteobacteria	13.900 ± 4.604	34.358 ± 2.901	0.01
Spirochaetota	6.544 ± 1.704	0.186 ± 0.036	0.005
Porphyromonadaceae	25.328 ± 4.148	10.671 ± 2.892	0.02
Flavobacteriaceae	2.465 ± 0.984	8.788 ± 1.762	0.01
Peptostreptococcaceae	6.767 ± 0.824	3.280 ± 0.797	0.03
Fusibacteraceae	5.448 ± 1.175	1.739 ± 0.477	0.03
Spirochaetaceae	6.536 ± 1.704	0.185 ± 0.036	0.005
Weeksellaceae	0.595 ± 0.261	5.599 ± 2.121	0.01
Xanthomonadaceae	0.439 ± 0.293	5.057 ± 1.765	0.01
Neisseriaceae	0.916 ± 0.324	2.909 ± 0.545	0.02
*Porphyromonas*	25.328 ± 4.148	10.671 ± 2.892	0.02
*Fusibacter*	5.448 ± 1.175	1.739 ± 0.477	0.03
*Treponema*	6.504 ± 1.696	0.176 ± 0.036	0.005
*Bergeyella*	0.413 ± 0.229	5.222 ± 2.128	0.03
*Flavobacterium*	0.422 ± 0.367	5.058 ± 1.697	0.01
*Luteimonas*	0.419 ± 0.294	4.718 ± 1.790	0.02

CON, cats fed with a basal diet; CPG, cats received the basal diet with a post-sprayed composite probiotic formulation. Values are mean ± SEM (*n* = 6). Statistical significance was determined at *p* < 0.05.

## Data Availability

The data supporting the findings in this research are available by contacting the corresponding author on request.
